# Conductive Layers on a Shrinkable PET Film by Flexographic Printing

**DOI:** 10.3390/ma15103649

**Published:** 2022-05-20

**Authors:** Sandra Lepak-Kuc, Katarzyna Wasilewska, Daniel Janczak, Tatiana Nowicka, Małgorzata Jakubowska

**Affiliations:** 1 Institute of Metrology and Biomedical Engineering, Faculty of Mechatronics, Warsaw University of Technology, Sw. Andrzeja Boboli 8, 02-525 Warsaw, Poland; katarzyna.wasilewska.dokt@pw.edu.pl (K.W.); daniel.janczak@pw.edu.pl (D.J.); tatiana.nowicka.dokt@pw.edu.pl (T.N.); maljakub@mchtr.pw.edu.pl (M.J.); 2 Masterpress S.A., Jacka Kuronia 4, 15-569 Białystok, Poland; 3 Centre for Advanced Materials and Technologies (CEZAMAT), Warsaw University of Technology, 02-822 Warsaw, Poland

**Keywords:** shrinkable PET film, printed electronic, shrink sleeve labels, conductive inks

## Abstract

In this study, the extremely important and difficult topic of flexographic printing on a heat-shrinkable substrate was taken up. Six commercially available, electrically conductive inks based on silver, copper and graphite nanoparticles were selected and tested upon their applicability for printing on the temperature-sensitive PET material. As a printing substrate, the one-direction heat-shrinkable PET film, with a maximum shrinkage of 78%, was selected. All of the examined inks were subjected to the printing process throughout three different anilox line screens. The tested inks, along with the electric paths printed with them, were subjected to various tests. The main parameters were evaluated, such as printability combined with the rheology tests and ink adhesion to the examined PET substrate together with the electrical conductivity before and after the shrinkage.

## 1. Introduction

The market for all kinds of labels attached to packaging is growing at a rate of 6% annually, and the main growth driver is the development in the segment of smart and custom labels [1,2]. The main advantage of intelligent packaging is to engage the customer with the product through the retail display or the packaging itself, thereby strengthening the purchasing decision. Combining this fact with manufacturers’ drive to increase sales points to a growing need to develop decorative and functional labels that provide flexibility and can be processed at high speed using traditional printing methods, such as flexographic printing [3].

As of today, smart labels primarily concern temperature indicators that for example, change colour to convey information to the consumer about how fresh a food product is. However, the trend is developing fast, with more and more manufacturers interested in radio-frequency identification (RFID) tags for checking the expiry date. Easier electronic control over product batches would be thus saved and readable [4,5,6]. A barrier is often encountered here. The necessary stage of producing such labels is to create an electrically conductive layer on the label, and the substrate is challenging in terms of such layers, being a heat-shrinkable polymer. Such shrinkable films are indeed extremely useful materials in the packaging industry. Some of their most important uses are shrinkable food packages, multipacks and labels [7]. There are two types of shrink materials on the market, the former of which shrinks in a multidirectional manner and the second one only in one specific direction, transverse direction (TD). TD shrinkable films have found applications, such as so-called shrink sleeve labels (SSL), which exhibit many advantages when compared to the standard self-adhesive labels (SAL). SSLs can take the shape of a container and have more space for printing [8], which leads to better cost-effectiveness in the decoration of highly complex-shaped containers. Additionally, the use of SSL may enable the reduction of the wall thickness of plastic and glass containers and the elimination of the need for coloured containers. Sleeve labelling has been predominantly used for consumer goods packaging, for example, food and beverage products, cosmetics and toiletries, healthcare and pharmaceutical supplies and household care products [7,8]. Consumer packaging will most definitely directly influence the growing global demand for intelligent packaging. Production of a variety of intelligent packaging has gained increasing attention in the last decade. The functionality and properties of this type of packaging entail increasing usage of printed electronic techniques [3].

Packaging labels today are manufactured with various printing techniques [9]. Among other techniques, flexographic printing has the highest economic potential, due to a combination of its high throughput and low-cost production, which makes this technique widely used in the packaging industry [10,11,12]. The printing process is based on an anilox roller and polymer plate. An anilox roller is a cylinder in which microscopic cells are engraved. Anilox rollers are defined by capacity, given in cm^3^/m^2^, which depends on the shape and depth of these cells. The highest points on the polymer plate create a graphic artwork which is printed during the process. In the flexographic method, ink is transferred at first from the anilox roller to the polymer plate and next from the polymer plate to the printing substrate. The amount of ink which is transferred onto the substrate varies normally between 0.25 and 6 µm and depends mostly on the capacity of the anilox roller and ink viscosity [13].

The usage of printing techniques also enables a successful production of electronic circuits on a variety of substrates, starting from rigid wafers [14], through rigid and flexible laminate plates [8,15], various types of metal and polymer foils [16], ending on stretchable substrates [17], which makes those techniques perfect candidates for the successful production of smart labels. Labelling solutions with integrated electronic systems can already be found in several patent applications [18,19]. However, in all the published solutions, electronic circuits are manufactured on substrates that do not have heat-shrinkable properties. Printing of electronic elements on shrinkable substrates according to the current state of knowledge is unsolved and it is undoubtedly one of the most technologically difficult problems. One of the basic challenges that must be faced to produce electronic components on a heat-shrinkable label is the selection of the appropriate conductive ink. This issue is not trivial to solve due to some requirements such ink has to fulfil, such as chemical compatibility with the PET heat-shrinkable substrate, high flexibility during the shrinking process to avoid the ink cracking during the application process and an extremely low application temperature. Shrinkable substrates require low temperature and a fast curing process; otherwise, materials will constrict. Both in the offer of ink manufacturers and the scientific literature, it is a challenge to find any electrically conductive materials dedicated to heat-shrinkable substrates, and the requirement to match the flexographic technology is an additional complication. However, there are reports of conductive inks suitable for low-temperature sintering, being most commonly materials based on silver nanoparticles and achieving different electrical resistivity values of 9.9 × 10^−8^ Ω·m [20]; 1.26 × 10^−5^ Ω·m [21]; 1.2 × 10^−7^ Ω·m [22]. Moreover, nowadays a range of solvent-based pastes and inks with low curing temperatures, usually not exceeding the value of 130 °C can also be found on the commercial market [23,24]. It should also be emphasized here that for most low-temperature inks, there is a relationship between the drying temperature and the drying time. The lower the drying temperature is, the longer the curing process required. When considering printing on delicate substrates, the family of UV-curable acrylic materials may also be worth considering. UV-curing conductive pastes typically consist of functional conductive micro- or nanoparticles, a carrier polymer, photoinitiators, diluents and, optionally, adhesion additives [24,25,26]. Publications indicate that the process of producing the UV-cured conductive layer, despite the original UV-sintering, often requires an additional heat treatment following the UV-involved drying. Temperatures that are mentioned to ensure the crystallisation of silver particles suspended in the paste form are even as high as 250 °C [26,27,28]. Both the technologies and the materials used are relatively young and often protected by proprietary patents [29,30,31]. Moreover, the trait of flexibility does not necessarily go hand in hand with a low sinter temperature. A key limitation in the use of all mentioned inks in the heat-shrinkable sleeve industry, may be the material’s non-susceptibility to substrate shrinkage, and such inks are very rarely subjected to shrinkage tests. In this work, an attempt was made to find a commercially available conductive ink compatible with the shrinkable PET substrate intended for the packaging market. Assessment of ink adhesion, printability, shrinkability and resistance of conductive ink printed on PET thermoshrinkable material was made. All these parameters are necessary to create the production process of PET shrink sleeve labels.

## 2. Materials and Methods

The 50 μm PET film from Klockner Pentaplast has been used as TD shrinkable material. Six different kinds of conductive inks were tested. The choice of commercial inks depended on the possible uses of these results in product labels, which are commercial solutions, and thus market availability is important. Table 1 presents a summary of the examined inks with information about the material of the functional phase, solvent and sheet resistivity specified by the supplier. All tested inks have been chosen based on both the drying process requirement of low temperature and the viscosities of these inks being suitable for the flexography printing method. In addition, according to technical specifications, all chosen inks are resistant to high temperatures after curing. This attribute is extremely important for the shrink sleeve label application on the container, as this application includes hot steam treatment. We chose mainly silver inks as this material of the functional phase dominates the market among the inks meeting the above requirements. For greater objectivity of research, we also searched the market for inks other than silver that could be used in heat-shrinkable labels; hence, the list also includes copper and graphite inks.

### 2.1. Methods

Within this work, samples have been printed by flexographic method to evaluate the properties of different conductive inks which were printed on shrinkable PET material. Inks themselves were subjected to rheology tests. Printed paths parameters were also measured.

#### 2.1.1. Printing Technique

The printed layers were designed to evaluate different properties of the printed samples, e.g., printability, ink adhesion, conductivity and shrinkability. The designed images were created on a polymer plate. Graphic artwork contains lines with 100% coverage. Printing trials were performed on a sheet-fed Testacolor 171 device produced by a company named NSM Norbert Schlafli AG. The printing press runs with a speed of 10 m/min. For the proper ink transfer from the anilox roller to the polymer plate, the doctor blade was used. Three different anilox rollers were used, which line screens and capacities are presented in Table 2.

**Figure 1:**
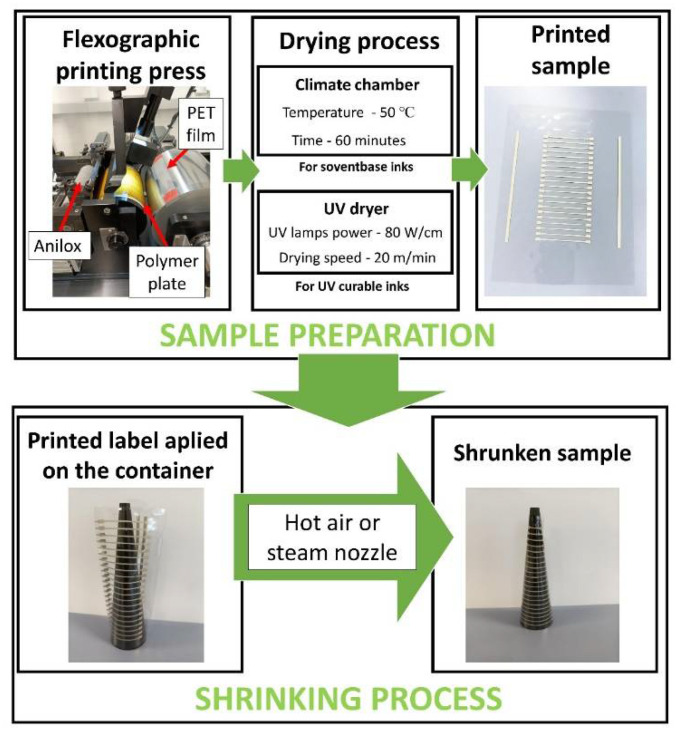
Scheme illustrating the stages of the preparation of the conductive paths on the shrinkable substrate.

The prints were dried offline. Samples that require UV drying were dried on an Aktiprint Mini 18-2 UV dryer. The drying speed was 20 m/min, and the power of UV lamps was 80 W/cm. Samples that require temperature for drying were dried in a Binder Drying Oven Type FD 115 climate chamber. The drying process lasted 60 min with a constant temperature of 50 °C. The stages of sample preparation were shown on Figure 1.

#### 2.1.2. Rheology

The inks’ shear viscosity was measured with the use of R/S Plus Rheometer (Brookfield Engineering, Middleboro, MA, USA) equipped with an RCT-50-1 spindle, dedicated to the viscosity ranging between 0.006–50,900 Pa·s. A shear rate from 0 to 400 s^−^^1^ for 100 s was used due to the limitation of the viscometer. The constant temperature of 25 ± 0.5 °C was kept during the measurements. The data was then analyzed using the Rheo 3000 software (Brookfield Engineering, Middleboro, MA, USA). The viscosities of the tested inks at the shear rate ~100 s^−^^1^ were compared.

#### 2.1.3. Electrical Conductivity

In this paper, conductivity was assessed by measuring the resistance on a specific section of the print, followed by conversion of the resistance to a square. A two-point conductivity measurement method was used. Conductivity was tested for 25 samples of each of the tested inks. The results reported are the average values.

#### 2.1.4. Ink Adhesion

Ink adhesion was tested with Tessa Tape 73422-620. This method is based on sticking the tape (Tessa Tape 73422-620) on a 5-cm section of a printed layer. The tape is peeled off after 1 min. Thereafter, a visual inspection of the ink residue on the tape is performed. If one does not observe ink fragments on the tape strip, it proves 100% adhesion of ink to the printing substrate. On the other hand, if the tape is completely covered with ink, it means that the ink adheres to the substrate by 0%. With this method, ink adhesion is assessed visually. A three-level evaluation scale was adopted:

1—good adhesion (100–90% ink residue on the printing substrate)
2—medium adhesion (90–40% ink residue on the printing substrate)
3—poor adhesion (40–0% ink residue on the printing substrate)

The ink adhesion was tested for 10 samples of each of the tested inks. The results reported are the average of the multiple measurements.

#### 2.1.5. Printability

Printability was assessed using an optical microscope Keyence VHX 6000 (Keyence Corporation, Osaka, Japan). The observations were made to assess the quality of the printed conductive path. The quality parameters observed were width and defects occurring in the structure of a printed path. Printability was observed for 5 samples of each of the tested inks.

#### 2.1.6. Shrinkability

For the examination of the ability of the printed layers to shrink, the samples were conducted to the shrinking process with the use of an appropriate metal profile allowing for a different percentage of material shrinkage resulting in 10%, 20%, 30%, 40% and 50% reduction of the length of the printed path. The printed layers of each of the tested inks were hardened at 50 °C for 60 min beforehand. The conductivity of printed tracks was measured and registered before the shrinking process. The dried prints were formed in heat-shrinkable sleeves and applied to the profile. Such prepared samples were then subjected to the action of heat which caused their shrinkage to the shape of the profile used. This container is profiled in such a way as to obtain the appropriate shrinkage at a specific height. The shrinkage percentage value was defined by differences in the circumferences on the particular height of the metal profile.

We examined two shrinkage mediums, which were the hot air and the steam. The hot air shrinking process was conducted on a climate chamber Binder Drying Oven Type FD 115 (Keyence Corporation, Osaka, Japan) at the temperature of 90 °C. The steam shrinking process was carried out with a steam nozzle with a steam temperature of around 90 °C. The pictures of the sample before and after the shrinking process are shown in Figure 1. After that process, the shrunk label was removed from the container, and conductivity was measured in places where shrinkage of the length of the printed paths amounted 10%, 20%, 30%, 40%, 50% respectively. Microscopic observations of the samples after the shrinking process were also carried out using a Keyence VHX 6000 optical microscope and a Hitachi 8230 (Hitachi High-Tech Co., Tokyo, Japan) scanning electron microscope.

## 3. Results and Discussion

### 3.1. Rheology Test

**Figure 2:**
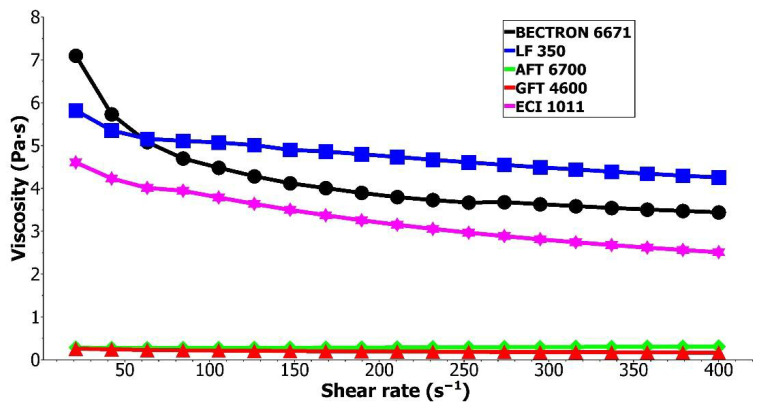
The viscosity of the tested inks.

Despite the selection of the printing substrate, all of the inks dedicated to the flexography technique must enable printing the paths. The printability of specific material depends on a couple of parameters. One of the most important in this matter is the ink’s viscosity. Flexography mostly requires rapidly drying inks. Most often, inks with a viscosity in the range of 0.1–0.5 Pa·s are indicated as the most suitable [13,32]. However, higher viscosities are also acceptable as long as the proper printing parameters are provided. The viscosity was measured for all inks tested. The results are shown in Figure 2. The figure omits the result for S2 due to the very high relative viscosity values in the range of several dozen Pa·s, as well as the lack of shear-thinning effect.

Despite all tested inks being suitable for the flexographic printing method, there are visible differences in their viscosities. The viscosities at the shear rate ~100 s^−1^ were 15.12 Pa·s for S2, 4.48 Pa·s for S1, 5.07 Pa·s for C1, 3.79 Pa·s for S4, 0.28 Pa·s for S3 and 0.22 Pa·s for G1. Despite this variety of ink viscosities, all of them enabled the flexographic printing of paths. However, graphite GFT4600 ink required additional adjustments, specifically, a close doctor blade system. It was caused by the quick drying of the ink, and the closed doctor blade system prevented the ink from drying on an anilox roll during the printing process. The variety of viscosities of inks adapted to flexographic printing may also indicate the compatibility with various types of substrates and a variety of applications. For such new research as presented in this article, testing inks with different viscosities is advisable to finally assess what viscosity of inks is predisposed to create conductive paths resistant to substrate shrinkage.

### 3.2. Electrical Conductivity Tests

Another parameter which is essential for producing an electrically functional label is the resistance of paths. Sheet resistance was measured for all tested inks printed with an anilox roll 25 cm^3^/m^2^ before the shrinking process (Table 3).

The best conductive properties are observed for S2 ink. For S4 and C1 inks, the electrical conductivity of paths dried at 50 °C was not measurable, which may result from the fact that the conducted drying temperature is lower than the ones recommended in the materials Technical Data Sheets (TDS). For the reliability of resistance measurements, prints were also made on a standard, non-shrinkable substrate, which was 125-μm PET film from Dupont Tejin Films, allowing drying paths at higher temperatures provided in TDS. Three inks were tested at higher drying temperatures, adjusted to the recommendations in the TDSs, two for which no measurable resistance value was obtained at 50 °C, and the most promising S2 ink, due to the fact that the recommended temperature for this ink is higher than 50 °C. For the non-shrinkable substrate, the resistance at the drying temperature of 50 °C was also tested to eliminate the influence of the substrate on the electrical resistance results. The results for paths dried at 50 °C measured on the non-shrinkable film are consistent with the results obtained for the tested shrinkable substrate. While increasing the drying temperatures to the ones recommended in TDS, which were 120 °C for S2 ink, 150 °C for S4 ink and 200 °C for C1 ink, only the silver-based inks showed an improvement in electrical parameters. The sheet resistance values of the 0.06 Ω/sq and 0.03 Ω/sq were measured, respectively, which are consistent with the manufacturers’ declarations. The measurable conductivity values for C1 ink were not obtained even in the drying process at 200 °C. Regardless of the possibility of achieving the required electrical conductivities for high drying temperatures, the target substrate is heat-shrinkable and therefore temperature-sensitive materials. Therefore, the priority was to select the drying process according to its thermal resistance, and such drying conditions were sufficient for the S2 ink.

A close correlation can be observed between the resistance and the amount of ink applied. Therefore, samples were prepared using three aniloxes differing in capacity and thus the amount of ink transferred to the printing substrate. An anilox with a capacity of 9 cm^3^/m^2^ had the lowest, while anilox 25 cm^3^/m^2^ had the highest ink transfer. The resistance was respectively 2.6 Ω/sq for anilox 9 cm^3^/m^2^ and 0.32 Ω/sq for anilox 25 cm^3^/m^2^. The tracks printed for the middle-size anilox, of the capacitance 12 cm^3^/m^2^ had a resistance of 0.5 Ω/sq. This is understandable as the more ink is transferred, the more conductive particles are contained in the layer. This fact is widely described in the literature, so obtained results follow the rule [33,34].

Electrical conductivity is a fundamental property required of printed tracks. The lowest resistivity, meaning the best electrical parameters, are achieved while using the high transfer anilox 25 cm^3^/m^2^. Accordingly, the remaining tests described below were carried out on the layers obtained using this anilox.

### 3.3. Printability Tests

**Figure 3:**
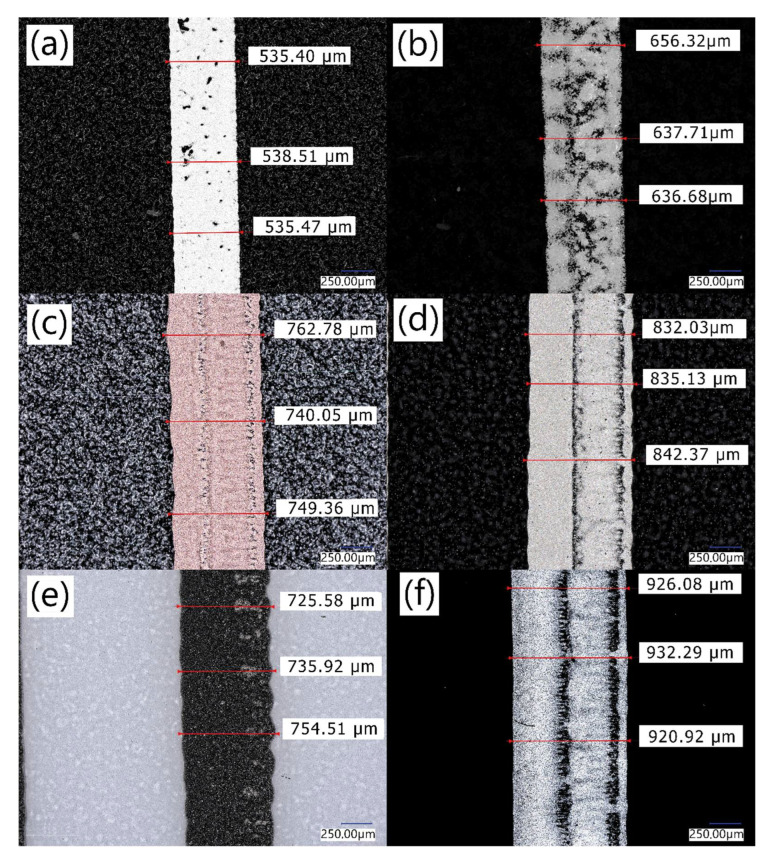
Microscope pictures of printed paths using (**a**) S2, (**b**) S1, (**c**) C1 (**d**) S3 (**e**) G1, (**f**) S4 inks.

In order to fully assess the printability of inks, paths were printed and dried for all of the tested materials, and the samples were subjected to microscopic observations (Figure 3).

As shown in Figure 3, different inks exhibit different printability on the thermo-shrinkable PET film. Defects can be observed for each of the inks, but their number and dimensions vary widely from material to material. The worst quality print had S1 ink (Figure 3b). It results in significant areas without conductive ink. That leads to a lack of conductivity. The best continuity of the printing, understood as the smallest amount of non-inked spots in the image area can be observed for S2 and G1 inks. Such a lack of defects on the printed surface is especially important in the case of electrically conductive inks as any break in the printed path can cause a reduction in conductivity. Based on the print setup parameters, all of the paths should have the same dimension, which is 500 µm. However, despite using the same polymer plate, the analysis of the microscope pictures points to differences between the width of printed patches. The dimension disagreement is caused by the differences in viscosity and surface tension of printed inks as well as process parameters, such as low printing speed and offline drying process. Longer time, measured in minutes, between printing and drying processes allowed inks with lower viscosity to split more on a substrate. That phenomenon leads to the width deviations. The biggest width deviations have been noticed for S4 ink (Figure 3f), and the best result was achieved by S2 ink. According to Figure 3 the S2 ink, which is the ink with the highest viscosity measured, has the best printability properties on the thermo-shrinkable PET substrate. This may indicate that high ink viscosities are required for these types of substrates.

### 3.4. Ink Adhesion Tests

**Figure 4:**
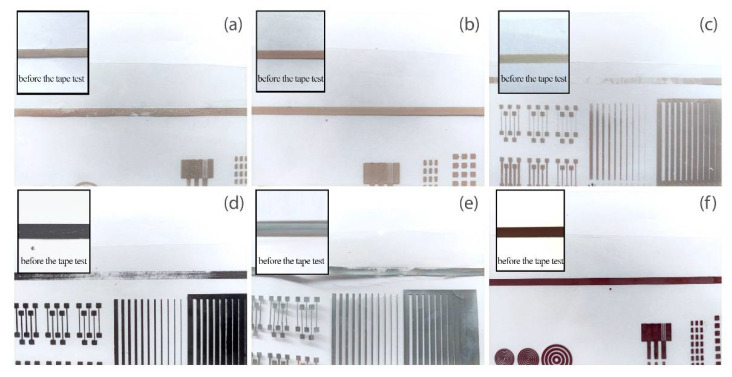
A picture of the layers subjected to the Tessa Tape adhesion test for (**a**) S1, (**b**) S2, (**c**) S3, (**d**) G1, (**e**) S4 and (**f**) C1 inks. The test was conducted on the straight layer at the top of the printed pattern.

When considering the usage in SSL labels, the adhesion of prints is an extremely important parameter. The adhesion measured for tested inks is presented in Table 4 and Figure 4. Poor adhesion of the printed layer to the printing substrate may cause detaching of the ink during the application of the label to the container [35]. As a result, graphic gaps would be visible in the final packaging and while considering electrically conductive inks, such gaps would result in a lack of electrical connection.

Results indicate that the best adhesion to the PET substrate was observed for S2 and C1. For S4 and S1, adhesion is slightly lower and included in the medium adhesion level. The two inks of the lowest viscosities, namely S3 and G1 are characterized by low adhesion, under 40%. The situation when the ink does not adhere to the substrate entails its crumbling during the shrinking process, which eventually results in gaps between the functional particles and a lack of conductivity. If the conductive path is damaged, the electronic system simply will not work. As a result, the label with the electronics circuit printed with low adhesion conductive ink will not fulfil its functionality. Although the ink adhesion test is a critical parameter for shrink sleeve labels and inks have to meet the adhesion requirements, only S2 and C1 inks can be categorized as useful on the SSL labels.

Considering the good printability, the best adhesive properties and low path resistance, the S2 ink has proved to be the most suitable for the studied application, and therefore, this ink was chosen for further research.

### 3.5. Shrinkability Tests

**Figure 5:**
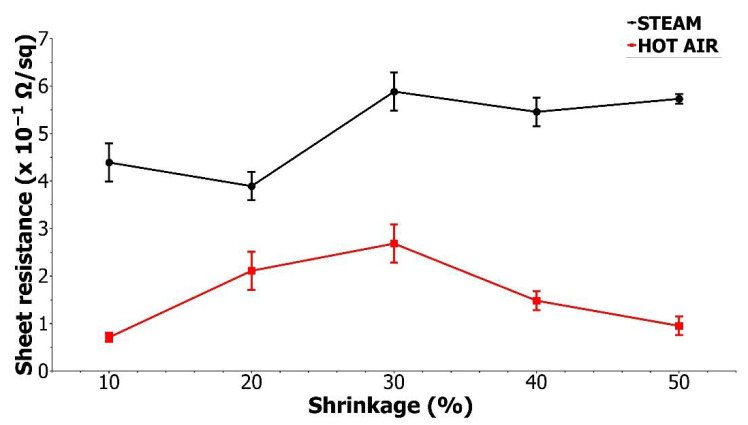
The sheet resistance of shrunken samples; whiskers indicate the standard deviation.

The next test carried out was the shrinkability test. The shrinking process was carried out using two different heating media which were hot air and steam. The sheet resistance was measured after the shrinkage, and the results are shown in Figure 5.

**Figure 6:**
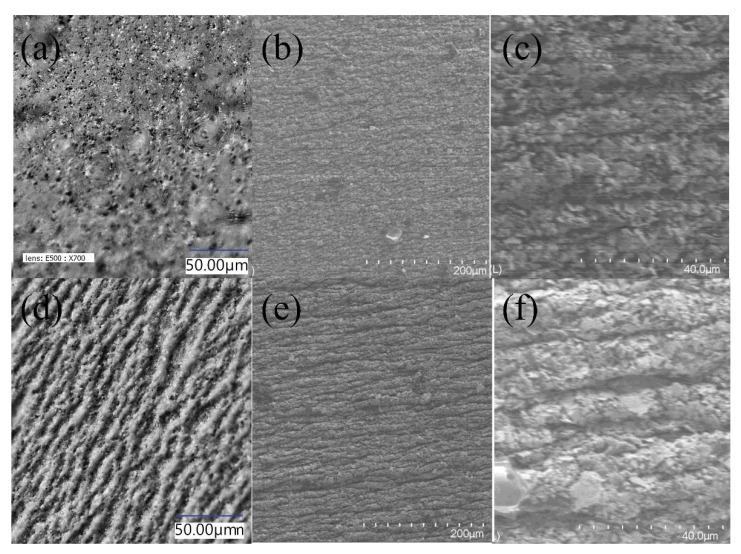
Images from the optical (**a**,**d**) and from the scanning electron (**b**,**c**,**e**,**f**) microscopes of the conductive path from S2 ink after shrinking by the hot air process (**a**–**c**) and by the steam process (**d**–**f**).

There is a visible difference between the resistance of the samples shrunken by hot air and steam. The differences are most probably caused by the mechanism of the shrinking process in different environments. The mechanism of a shrink sleeve label application is based on shrink sleeve film clamping to shrink the packaging thoroughly. The better the uniformity of the hot medium supply is, the better quality of distribution of the film compressive force is achieved. This prevents deformation of the printed film as well. Therefore, the best quality of the final label is provided [7,36] In a hot air environment, printed conductive inks shrink much slower. The hot air surrounds the samples evenly on all sides, and the shrinking process is slow and gentle. In case of nozzle steam shrinking, the hot steam is directed pointwise at the sample. The mechanism of intense material shrinkage point by point leads to cracks in the paint film and, consequently, to a reduction in electrical conductivity. Mentions about a similar behaviour of printed inks also appear for non-conductive graphic inks printed on a heat-shrinkable substrate [8,35]. The differences in the microscopic images of the surface structures of printed paths after the steam and hot air shrink processes were also noticeable (Figure 6).

In the optical microscope images, we observed a wrinkled surface for steam shrunken paths (Figure 6d) and a smooth surface of a layer shrunken by hot air (Figure 6a). In SEM images, a much smoother surface is also observed for the paths shrunk by the hot air method. In higher magnification images, it is possible to see the bulges also for hot air shrink specimens (Figure 6c), but they are much smaller than for steam nozzle shrinkage (Figure 6f). Such differences explain the lower resistance after the hot air shrinking process. The resistivity after the shrinking process was even lower than the one measured directly after the printing process. Such an improvement in electrical parameters may be related to better drying of the samples and thus more complete evaporation of solvents from the ink, but also may result from the condensation of prints during shrinking.

The conducted research clearly shows the advantage of the hot air drying process, in the case of applications for electrically conductive heat-shrinkable labels, with the use of selected silver ink of high viscosity.

### 3.6. Application as a Smart Label

**Figure 7:**
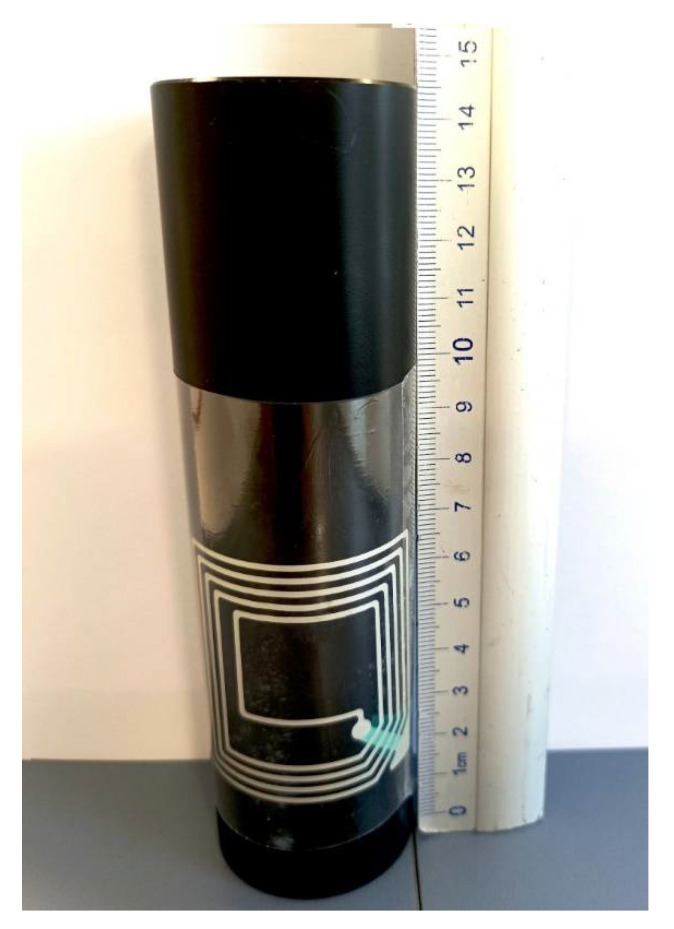
NFC antenna printed on shrink sleeve label applied on the container.

The examination and analysis of all parameters for conductive paths printed on the PET film, such as electrical resistance, printability, adhesion to the substrate and stability after the shrinkage conducted in both high-temperature and high-humidity environments, enabled the selection of the most suitable ink and successful production of the conductive tracks on a heat-shrinkable substrate. This substrate is dedicated to a specific application, namely for product labels on packages of various shapes. This is a major achievement as it can allow smart labels to be produced on these types of difficult packages. To confirm such a possibility, with the use of the selected ink, a near field communication (NFC) antenna was produced using the flexographic method directly on the heat-shrinkable label placed on the product packaging (Figure 7).

## 4. Conclusions

Six commercial conductive inks dedicated to flexographic technology have been tested in the context of their use on a heat-shrinkable substrate. Differences in their viscosities have been observed; adhesion to the tested substrate, printability and the obtained layer resistance have been assessed. Some of the tested inks, namely S4 and C1 inks, despite the good conductivity of the paths, dried at temperatures above 100 °C and showed a lack of conductive properties after a drying process concordant to the high sensitivity of the heat-shrinkable substrate temperature. Our research indicated the S2 ink as the best one among the tested inks. This ink characterizes the best adhesion to the printing substrate together with the low sheet resistance of 0.32 Ω/sq. This ink is characterised by the highest viscosity of all tested inks and high viscosity in the flexographic printing process causes increased ink transfer to the printing substrate, which may entail the lower resistance of layers. Further, S2 ink has very good printability properties. The dimension of the printed path which is 536 µm is very close to the element on the polymer plate which is 500 µm. The edges of the printed path are sharp and not wavy as it was in the case of the other tested inks. S2 ink also shows good conductive properties after the shrinking process. A smoother layer structure is observed after shrinking in hot air, which influences conductive properties. The observed increase in the electrical conductivity of the layers for the hot air shrinking process leads to the conclusion that this medium is suitable for heat-shrinkable labels with electrical elements. The presented research proves the possibility of the application of the conductive layers directly on a thermosensitive flexible substrate.

The presented studies represent a huge step forward in the matter of printing electrical circuits and electronic devices like sensors and NFC or RFID antennas, directly onto a shrink sleeve label. Smart packaging can change the way retailers, brand owners and consumers interact with products by communicating, engaging with customers, managing inventory systems and more. Brand owners and packaging manufacturers are looking for new functionalities to distinguish their products. Smart labels enable recognition at the point of sales and introduce interaction with the customer through packages that light up, as well as brand protection through the use of NFC labels. Over the next decades, the Organic and Printed Electronics Association (OEA) expects fully printed NFC and RFID labels, ambient intelligence and complex fully printed and integrated packages, including power source and storage, display, logic and sensors [3]. This means there is a greater demand to develop materials along with decorative labels that provide flexibility and can be processed at high speeds using printing methods like flexographic. We can predict that the presented results will develop intelligent packaging technologies and highly facilitate and accelerate obtaining fully printed NFC and RFID labels.

## Figures and Tables

**Table 1:** Tested conductive inks.

Ink	Commercial Name	Supplier	Conductive Particles	Solids Content [%]	Solvent	Sheet Resistivity (According to TDS)
S1	Bectron 6671	Elantas	silver	98	acrylic resin	<30 mΩ/sq/mil
S2	Bectron 6680	Elantas	silver	77	solvent based	<25 mΩ/sq/mil
C1	LF-350	Copprint	copper	85	solvent based	<0.004 Ω/sq/25 µm
S3	AFT6700	Sun Chemical	silver	>50 <80	water based	<15 mΩ/sq/mil
G1	GFT4600	Sun Chemical	graphite	Data not available	water based	<100 Ω/sq
S4	ECI1011	Henkel	silver	75.6	solvent based	<0.005 Ω/sq/25 µm

**Table 2:** Aniloxes used for printing.

Anilox Line Screen [L/cm]	Anilox Capacity [cm^3^/m^2^]
80	9
140	12
40	25

**Table 3:** The sheet resistance of printed conductive inks.

Ink	Sheet Resistance [Ω/sq]	Standard Deviations
S1	5.18	0.48
S2	0.32	0.07
S3	2.65	0.35
G1	45.3	4.28
S4	No conductivity properties	-
C1	No conductivity properties	-

**Table 4:** The adhesion of the layers printed with the usage of tested inks (1—good adhesion, 2—medium adhesion 3—poor adhesion).

Tested Ink	Adhesion of the Print
S1	2
S2	1
S3	3
G1	3
S4	2
C1	1
